# Supplementary material for: Testing an optimally weighted combination of common and/or rare variants with multiple traits
Source: PLoS One. 2018 Jul 26;13(7):e0201186. doi: 10.1371/journal.pone.0201186 (PMC6062080; doi:10.1371/journal.pone.0201186)
Supplement: S1 File — (PDF) [file pone.0201186.s001.pdf]

Supplementary Information

Testing an optimally weighted combination of common and/or rare variants with multiple traits

Zhenchuan Wang[1], Qiuying Sha[1], Shurong Fang[2], Kui Zhang[1], Shuanglin Zhang[1,*]

[1]Department of Mathematical Sciences, Michigan Technological University, Houghton, Michigan, United States of America

[2]Department of Mathematics and Computer Science, John Carroll University, University Heights, Ohio, United States of America

[*]Corresponding author

Email: shuzhang@mtu.edu

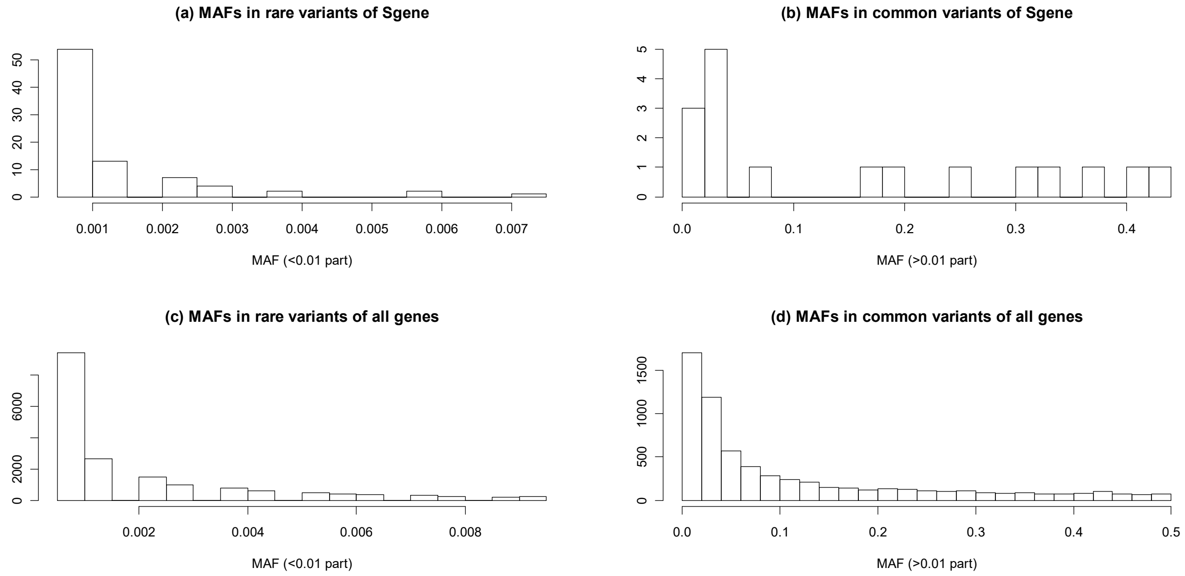

Figure A. The distributions of MAFs in the 100 variants in the Sgene and in the 24,487 variants in all of the 3205 genes. Figure (a) gives the histogram of MAFs in rare variants (MAF<0.01) in the Sgene and Figure (b) gives the histogram of MAFs in common variants (MAF>0.01) in the Sgene. Figure (c) gives the histogram of MAFs in rare variants (MAF<0.01) in all of the 3205 genes and Figure (d) gives the histogram of MAFs in common variants (MAF>0.01) in all of the 3205 genes.

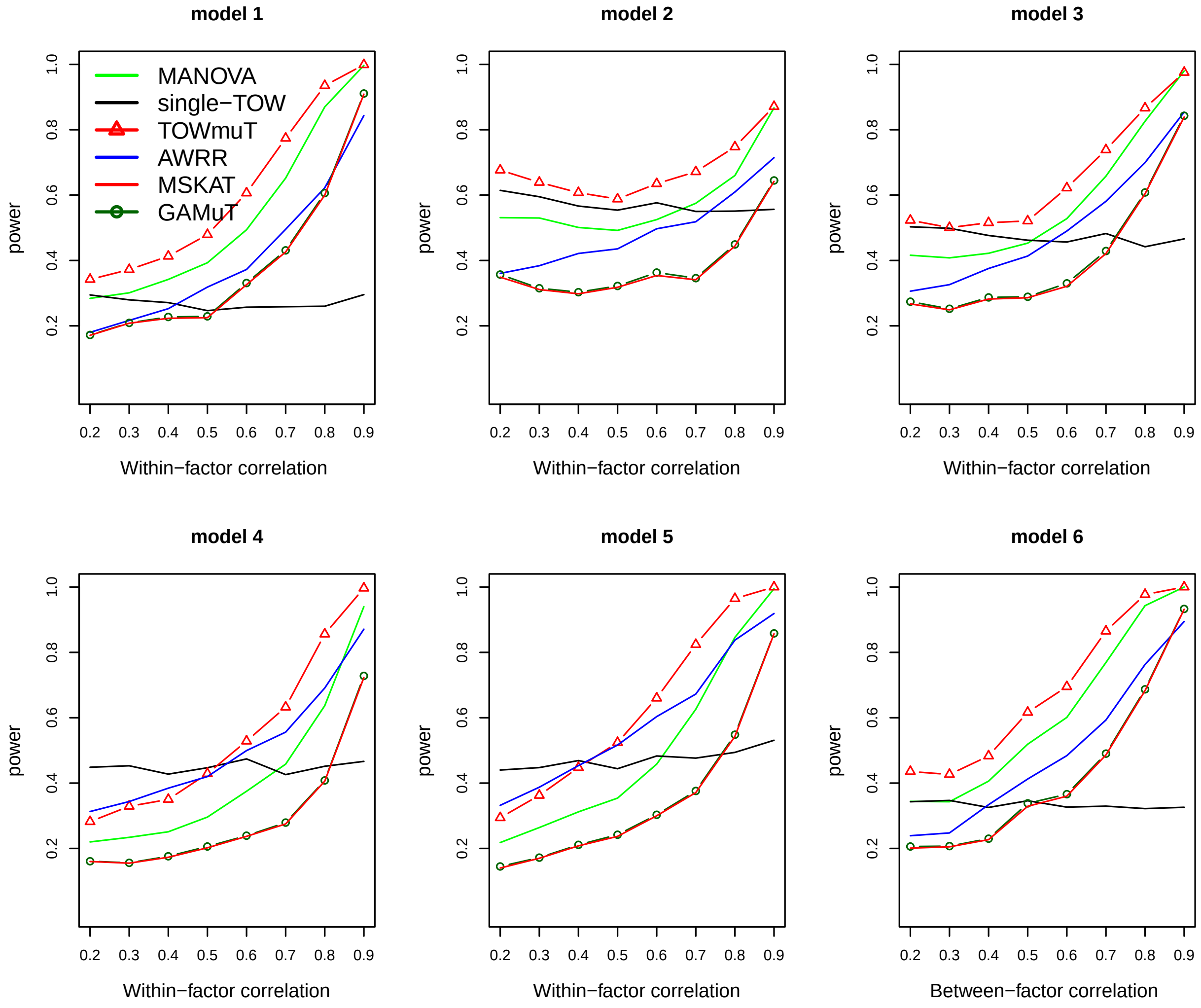

Figure B. Power comparisons of the six tests (Single-TOW, MSKAT, AWRR, MANOVA, GAMuT and TOWmuT) for the power as a function of within-factor correlation for models 1-5 and between-factor correlation for model 6 for 10 quantitative traits with covariates. The sample size is 1000. The percentage of the causal variants is 0.2. All causal variants are risk variants and $\rho = 0.5$ is for models 1-5. Heritabilities for models 1-6 are 0.05, 0.09, 0.08, 0.03, 0.03, and 0.06, respectively.

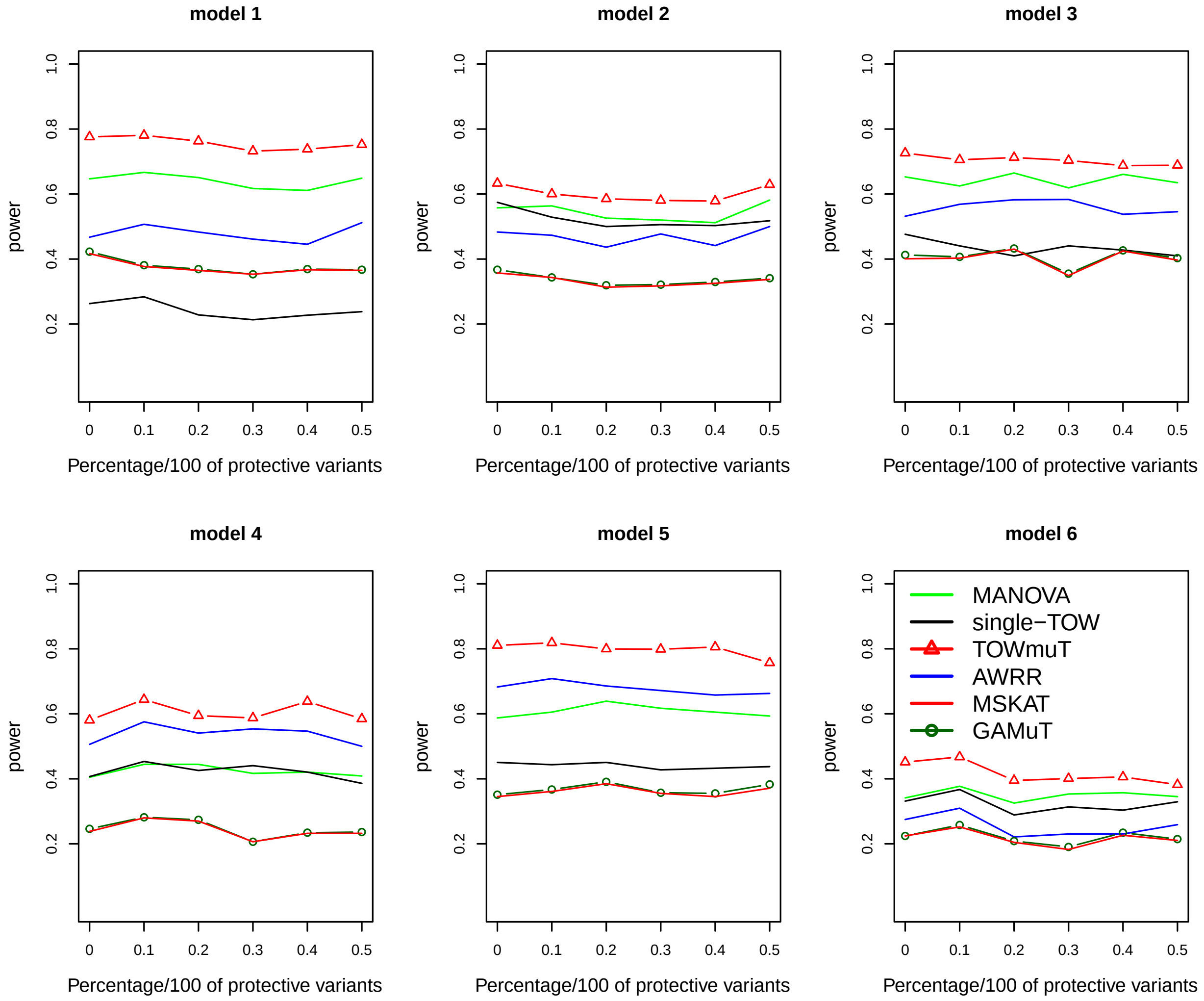

Figure C. Power comparisons of the six tests (Single-TOW, MSKAT, AWRR, MANOVA, GAMuT and TOWmuT) for the power as a function of the percentage/100 of protective variants among causal variants for 10 quantitative traits with covariates. The sample size is 1000. The percentage of the causal variants is 0.2. The between-factor correlation is 0.3 and the within-factor correlation is 0.7.

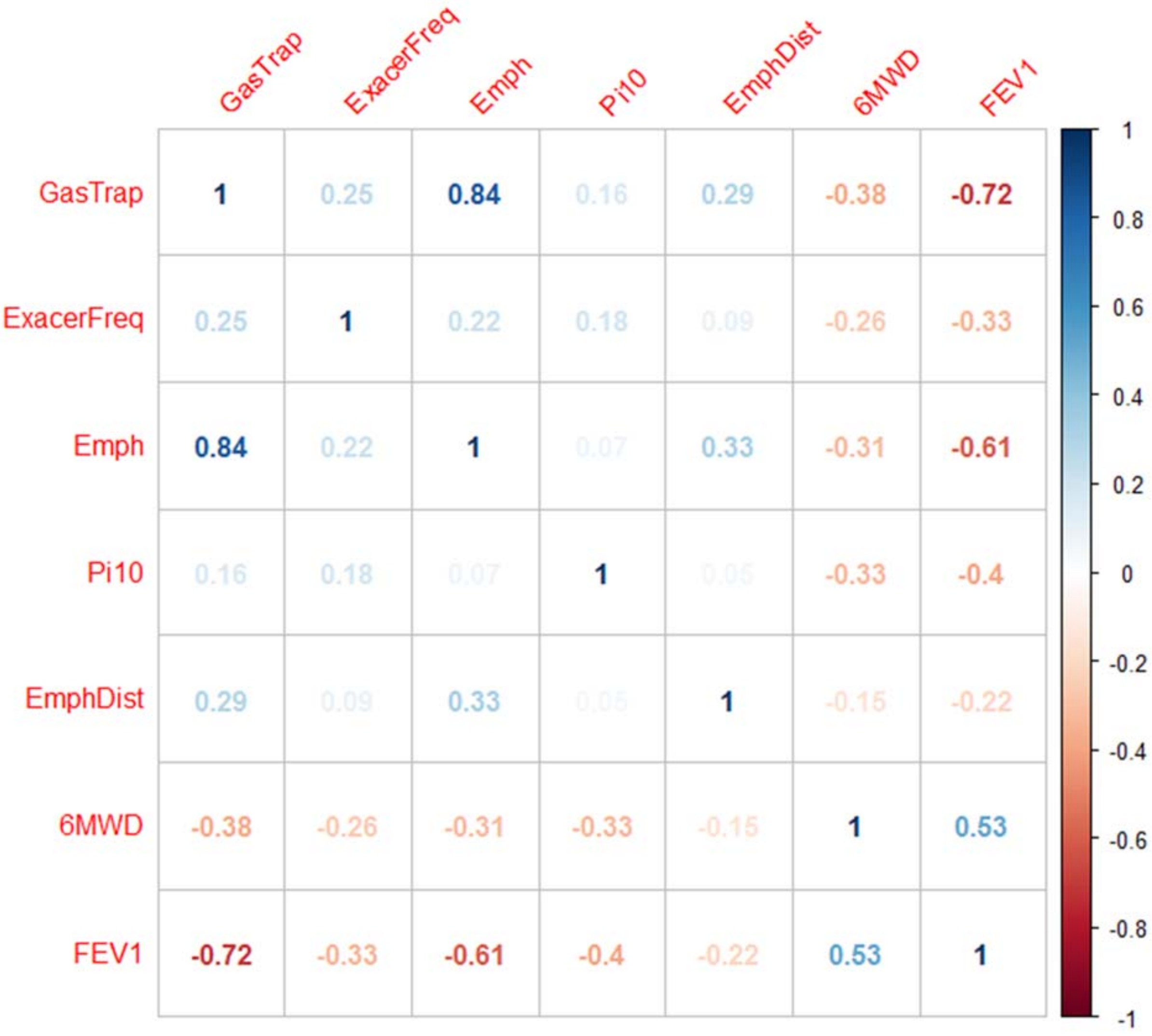

Figure D. The correlation matrix plot of the seven COPD-related phenotypes.
